# The American Medical Associaton, and Its Presidency

**Published:** 1874-02-15

**Authors:** 


THE AMERICAN MEDICAL ASSOCIATION, AND ITS PRESIDENCY.—"In view of the coming meeting, the question very naturally comes up, and is frequently asked, who will the profession of *this State* put forward as their choice for the presidency, in case the Association tenders to it the *usual* compliment of electing its president from the State in which it convenes?"

We clip the above from an editorial in the *Peninsular Journal of Medicine* for January; and it, together with what follows it in the next paragraph of the article, shows clearly that our brethren in Michigan are in danger of committing the old error of assuming that, because the Association is to meet in Detroit, the presidency must belong to them. And the article from which we have quoted gives unmistakable evidence that they are already organizing their factions, and laying the foundation for a nice home quarrel.

We would suggest to our friends in the Peninsular State that they save themselves all such trouble, by remembering that the Association has not elected a president residing in the city or State where they were holding the meeting, for the last eight or ten years; consequently, there is no such "*usual compliment*" as alluded to in the above paragraph.

And the most certain way to prevent such a *compliment* from being tendered for ten years to come, is for the profession in each locality to get well by the ears with each other before the time of the meeting.

